# Use of text-mining methods to improve efficiency in the calculation of drug exposure to support pharmacoepidemiology studies

**DOI:** 10.1093/ije/dyx264

**Published:** 2018-02-06

**Authors:** Stuart McTaggart, Clifford Nangle, Jacqueline Caldwell, Samantha Alvarez-Madrazo, Helen Colhoun, Marion Bennie

**Affiliations:** 1 Public Health and Intelligence Strategic Business Unit, NHS National Services Scotland, Edinburgh, UK; 2 Strathclyde Institute of Pharmacy and Biomedical Sciences, University of Strathclyde, Glasgow, UK; 3 Institute of Genetics and Molecular Medicine, University of Edinburgh, Edinburgh, UK; 4 Farr Institute of Health Informatics Research, Edinburgh, UK

**Keywords:** Text mining, natural language processing, dose information, prescriptions

## Abstract

**Background:**

Efficient generation of structured dose instructions that enable researchers to calculate drug exposure is central to pharmacoepidemiology studies. Our aim was to design and test an algorithm to codify dose instructions, applied to the NHS Scotland Prescribing Information System (PIS) that records about 100 million prescriptions per annum.

**Methods:**

A natural language processing (NLP) algorithm was developed that enabled free-text dose instructions to be represented by three attributes – quantity, frequency and qualifier – specified by three, three and two variables, respectively. A sample of 15 593 distinct dose instructions was used to test, validate and refine the algorithm. The final algorithm used a zero-assumption approach and was then applied to the full dataset.

**Results:**

The initial algorithm generated structured output for 13 152 (84.34%) of the 15 593 sample dose instructions, and reviewers identified 767 (5.83%) incorrect translations, giving an accuracy of 94.17%. Following subsequent refinement of the algorithm rules, application to the full dataset of 458 227 687 prescriptions (99.67% had dose instructions represented by 4 964 083 distinct instructions) generated a structured output for 92.3% of dose instruction texts. This varied by therapeutic area (from 86.7% for the central nervous system to 96.8% for the cardiovascular system).

**Conclusions:**

We created an NLP algorithm, operational at scale, to produce structured output that gives data users maximum flexibility to formulate, test and apply their own assumptions according to the medicines under investigation. Text mining approaches can provide a solution to the safe and efficient management and provisioning of large volumes of data generated through our health systems.

**Key Messages**

- A natural language processing (NLP) algorithm was developed to enable free-text dose instructions from 458 227 687 prescriptions of the NHS Scotland Prescribing Information System (2009–15) to be represented as quantity, frequency and qualifier.
- The final algorithm after clinical validation generated an overall structured output of 92.3%, which varied by therapeutic area (from 86.7% for the central nervous system to 96.8% for the cardiovascular system).
- Researchers can request a free-text dose instruction translated output as part of their PIS data extract for studies of systemic therapies through the eDRIS service run by NHS National Services Scotland [www.isdscotland.org/Products-and-Services/eDRIS/].

## Introduction

As health systems become more digitized, the volume and complexity of information grow rapidly and place demands on data providers to adopt new approaches to manage and provision these data in a form that promotes safe, effective and efficient use by stakeholders. Medicines are the most frequently used health technology, accounting for a rising proportion of health care budgets, and their impact is of interest to patients, clinicians, manufacturers and payers.^1^ The consequence is an ever-increasing demand to examine how medicines are being used in routine clinical practice, against a reducing evidence base as the medicines regulation landscape responds to growing public pressure for accelerated access through the concept of ‘adaptive licensing’.^2^

Drug utilization and pharmacoepidemiology studies seek to address this by better understanding how we use medicines in routine care and their effects, intended and unintended.^3^^–^^5^ Critical to this endeavour is a requirement for access to quality data on individual drug exposure across populations.^6^ The challenge is that most electronic prescribing systems permit prescribers to record dose instructions as free text, not structured data. The consequence is an extensive use of researchers’ time deployed to transform these data into a usable format by variable methods, often poorly documented, to calculate drug exposure.^7^ One solution is the application of rule-based natural language processing (NLP) methods to rapidly generate valid structured variables from free text, enabling drug exposure periods to be constructed consistently and reproducibly. NLP methods offer the ability to extract structured or standardized information from free texts in large volumes by defining sets of rules and lexicons in an iterative process.^8^

This approach is not new, with early applications often seeking to identify the presence of medicine prescribing attributes within clinical notes^9–11^ but not permitting calculation of drug exposure time periods. Shah *et al.*^12^ reported the application of a simple algorithm to a research database to codify free-text dose instructions to generate a derived daily dose. Karystianis *et al.*^7^ highlighted that the adoption of the Shah approach limited the ability of researchers to understand potential important variability in dosage information (e.g. two tablets up to three times daily would generate a single average-value daily dose of three tablets). They designed and tested a model to represent the variability and flexibility in drug directions, including the concept of minimum and maximum values for drug dosage, frequency and interval.^7^ Our study builds on this evidence and reports the design, testing and routine adoption at scale of a zero-assumption approach to the codification of free-text dosage information applied to the National Health Service (NHS) Scotland Prescribing Information System (PIS).^13^

## Methods

### Data source

The PIS is an administrative database recording all NHS prescriptions prescribed, dispensed and reimbursed in the community in Scotland.^13^ The PIS records information for about 100 million prescriptions per annum, around 98% of which include a unique person identifier. General practitioner (GP) prescribing accounts for about 95% of records, and these include an electronic prescription message containing free-text dose instructions. Other health care professionals’ prescriptions are largely paper-based, from which dose instructions are not captured. As the NHS is the universally used health care system in Scotland, the PIS provides a comprehensive record of primary care prescribing for a population of 5.3 million.

The study dataset included all electronic prescription messages for systemic therapies from April 2009 to May 2015 (inclusive). Each preparation prescribed is identifiable by a unique code based on the therapeutic groupings of the British National Formulary (BNF).^14^ These were used to identify systemic therapies and exclude topical treatments, which generally do not include information about the quantity to be administered within the dose instructions. The free-text dose instructions were initially cleansed to remove potentially confidential or disclosive information. The dose instruction free texts were then stratified according to the frequency with which they occurred (i.e. ≥ 1000, 100–999, 10–99, 2–9 times or once only within the dataset).

#### Phase 1: Definition of attributes and algorithm development

In common with others,^7^^,^^12^ we recognized that free-text dose instructions could be represented by three attributes–quantity, frequency and qualifier–each of which is then specified by a set of variables. An initial review of the 1000 most frequently occurring dose instructions identified that quantity and frequency attributes could each be represented by three variables to define minimum and maximum values and unit or period of measure, whereas qualifier was represented by two Boolean variables indicating ‘as directed’ or ‘as required’ (Table 1). We applied NLP methods to extract structured output defined by those variables using the Ciao implementation of the Prolog general purpose programming language [http://ciao-lang.org]. This was chosen because of the ease with which it is possible to include grammar syntax within the executable program.^8^ Words not associated with quantity or frequency information were ignored using rules. These rules checked for defined phrases and tested whether there existed a variable-length phrase that failed to satisfy any rule used to identify quantity or frequency information (e.g. ‘dispense by instalment’, ‘dissolve sachet’).

**Table 1.** Structured Dose Instruction Model

Dose attribute	Description	Variables
Quantity	The amount to be taken in each dose and the unit of measure, e.g. 5–10 ml	Amount_min
		Amount_max
		Amount_unit
Frequency	The number of times within a period that a dose should be taken and the period of measurement, e.g. 2–3 times a day	Freq_min
		Freq_max
		Freq_unit
	The interval between doses, e.g. 4–6 hourly	Interval_min
		Interval_max
		Interval_unit
Qualifier	Boolean variables to indicate further qualification of dose or frequency	As_required
		As_directed

The 1000 dose instructions were then processed using the algorithm, and the structured outputs were inspected manually for completeness and correctness. The rules and lexicons were modified and extended with the aim of achieving structured output for at least 85% of the distinct dose instruction and an error rate <1%. The process was repeated for sequential aliquots of the next 1000 most frequent instructions, until all with a frequency ≥1000 had been processed. Finally, a random sample of 500 dose instructions from each of the lower-frequency strata was introduced and the process repeated. Retrospective checking was performed to ensure that algorithm changes did not have a negative impact on the output compared with previous versions.

#### Phase 2: Clinical validation and refinement

All free-text dose instructions with a frequency ≥1000, and a new random sample of 500 from each of the other strata, were processed by the algorithm. The dose instructions, their structured outputs and any untranslated instructions were split into files. Each file was manually assessed by at least two reviewers from the Farr Institute@Scotland Pharmacoepidemiology Group, which included pharmacists, medical clinicians and researchers. Reviewers were asked to identify any errors and propose expected structured output for untranslated instructions. This feedback was used to refine the algorithm further, with retrospective checking to ensure no impact on the previously correct structured outputs.

#### Phase 3: Application of the final algorithm

The finalized algorithm was used to process the free-text dose instructions for all prescriptions in the dataset by therapeutic grouping. Output measures were the number of prescriptions with a structured output produced plus the number in which an element of discretion was exhibited (i.e. a range in quantity or frequency, or the presence of a qualifier, e.g. ‘one or two to be taken 4-6 hourly as required’).

## Results

For the period April 2009 to May 2015, there were 544 783 687 prescriptions with an electronic prescription message record, of which 458 227 687 related to systemic therapies. Of these, 456 684 974 (99.67%) had free-text dose instructions within the message. These dose instructions were represented by 4 964 083 distinct free-text dose instructions. A total of 13 593 (0.27%) distinct free-text dose instructions occurred ≥1000 times, accounting for 405 743 493 (88.85%) of all prescriptions with a free-text dose instruction. A further 75 081 (1.5%) distinct dose instructions occurred between 100 and 999 times within the dataset and accounted for an additional 20 293 362 (4.44%) prescription items (Table 2).

**Table 2.** Number of prescriptions by dose instruction frequency (2009–15) in the NHS Scotland Prescribing Information system (PIS) dataset

Frequency of dose instruction free-text	No. of distinct dose instructions	Total no. of prescriptions
≥ 1000	13 593	405 743 493
100 to 999	75 081	20 293 362
10 to 99	839 322	21 959 830
2 to 9	1 175 767	5 827 969
1	2 860 320	2 860 320
Total	4 964 083	456 684 974

### 

#### Phase 1

Initial inspection of the 1000 most frequently occurring free-text dose instructions affirmed that these could be represented by three attributes and associated variables. We adopted a zero-assumption approach (i.e. we did not assume a minimum quantity or frequency of zero in the presence of an ‘as required’ qualification). The representation of dose frequency was, however, modified to differentiate between doses within a period (e.g. twice daily) and intervals between doses (e.g. every 6 hours) (Table 1). A quantity unit is often omitted within free-text dose instructions and, even when present, is often implicit in the posological dose form (e.g. ‘one [tablet] to be taken at night’). We therefore modified the rules and lexicon so that a quantity unit was only specified within the structured output when it would impart additional meaning (e.g. ‘mg’ or ‘ml’). Table 3 presents a selection of dose instruction texts and how these are represented within the structured model.

**Table 3.** Examples of dose instructions and their structured output

	Quantity			Frequency						Qualifier	
				Period			Interval				
Dose instructions	Min	Max	Unit	Min	Max	Unit	Min	Max	Unit	As	As
										required	directed
Take two tablets four times daily	2	2		4	4	Day					
A half to one tablet two to three times a day when required	0.5	1		2	3	Day				TRUE	
10 mg to be taken weekly	10	10	mg	1	1	Week					
Two with each meal	2	2		3	3	Day					
Take 2.5 ml twice a day	2.5	2.5	ml	2	2	Day				TRUE	
Half a tablet twice a day when required	0.5	0.5		2	2	Day				TRUE	
Two puffs 6-hrly prn	2	2	Puff				6	6	hour	TRUE	
One to three every day	1	3		1	1	Day					
One or two to be taken every 4 to 6 hours	1	2					4	6	hour		
One twice daily as directed	1	1		2	2	Day					TRUE

#### Phase 2

A total of 15 593 free-text dose instructions that comprised all 13 593 distinct instructions occurring ≥ 1000 times plus 500 from each of the other strata (Table 2) were reviewed. The algorithm produced structured output for 13 152 (84.34%) instructions, and reviewers identified 767 (5.83%) incorrect translations, giving an algorithm accuracy of 94.2%. Additionally, reviewers were able to provide interpretations for 48% of untranslated dose instructions that were used to refine the algorithm further. The most significant change was to differentiate between dose frequency within a period and specification of an interval between doses. Reviewers felt that a literal interpretation of, for example, ‘every 4 hours’ to mean six times per day, was likely to lead to overestimation of consumption. Additionally, ‘unit tests’ that automate the detection of errors introduced by algorithm changes were implemented.

The final algorithm comprised 23 high-level grammar rules to identify the three main dose attributes, with a further 217 rules that identified the values to populate the specific variable and information within dose instructions that could be ignored. These were supported by lexicons containing 1242 words and phrases, including spelling variants (Table 4).

**Table 4.** Types of rules in final algorithm

Type of rule	Description	Number
General	High level definite clause grammar rules e.g. one or two as reqd every 4 to 6 hours for pain breakthrough dispense weekly quantity qualifier frequency statement	23
Quantity	Rules to identify and interpret the quantity to be taken in each dose e.g. one or two minimum indicator of range maximum	34
Frequency	Rules to identify and interpret instructions to take according to a number of doses within a period e.g. 2–3 times daily minimum indicator of range maximum indicator of within period period unit	51
	Rules to identify and interpret instructions to take according to intervals between doses e.g. every 4 to 6 hours indicator of interval between minimum indicator of range maximum interval unit	19
Qualifier	Rules specific to identifying explicit or implicit instructions to take as required	10
	Rules specific to identifying explicit or implicit instructions to take as directed	14
Other	Rules to identify statements and other information that is not related to quantity, frequency or qualifier e.g. for pain breakthrough, dispense weekly	89
Lexicons	Rules used frequently throughout the program e.g. rules to identify numeric values whether expressed as numbers or text	388
	Keywords and their spelling variants e.g. daily, dialy, dailly	454
	Specific keyword combinations that could not be processed by the general rule sets e.g. 3 times daily 2.5 mls is equivalent to 2.5ml 3 times daily	400

#### Phase 3

Table 5 presents, by therapeutic area, the output of the final algorithm applied to all 458 227 687 prescriptions in the dataset. Overall, the algorithm generated structured output for 92.3% of prescriptions, but this ranged from 86.7% for central nervous system (CNS) drugs to 96.8% for cardiovascular system (CVS) drugs. The proportion of dose instructions that allowed a degree of discretion (i.e. a range in quantity or frequency, or the presence of a qualifier) was <1% for most therapy areas, but was much higher for those where drugs are often used to provide symptomatic relief: musculoskeletal (4.9%); gastrointestinal (5.2%); respiratory (6.7%); and CNS drugs (21.3%), which encompass pain management.

**Table 5.** Conversion of free-text dose instructions to structure output by therapeutic area

			Indicators of discretionary dosing		
Therapy area	Total *n*	Structured output *n* (%)	Variable quantity or frequency *n* (%)	As required *n* (%)	As directed *n* (%)
Gastro-intestinal	49 584 619	45 408 768 (91.6)	2 347 535 (5.2)	1 834 975 (4.0)	901 652 (2.0)
Cardiovascular	141 703 356	137 125 926 (96.8)	355 268 (0.3)	747 988 (0.5)	2 368 611 (1.7)
Respiratory	35 180 421	33 514 364 (95.3)	2 247 739 (6.7)	6 204 193 (18.5)	900 796 (2.7)
Central nervous system	110 933 206	96 126 189 (86.7)	20 467 821 (21.3)	10 161 652 (10.6)	1 523 445 (1.6)
Infections	25 519 072	24 168 539 (94.7)	32 542 (0.1)	52 829 (0.2)	106 848 (0.4)
Endocrine	42 447 731	38 465 014 (90.6)	54 597 (0.1)	163 604 (0.4)	3 462 277 (9.0)
Obsterics, gynaecology, urinary tract disorders	12 964 366	12 144 962 (93.7)	50 654 (0.4)	230 156 (1.9)	1 376 945 (11.3)
Cytotoxics and immunosuppressants	2 204 372	1 923 960 (87.3)	3250 (0.2)	529 (0.0)	37 945 (2.0)
Nutrition and blood	17 468 476	15 720 743 (90.0)	83 804 (0.5)	130 334 (0.8)	862 703 (5.5)
Musculoskeletal	20 222 068	18 518 168 (91.6)	906 495 (4.9)	970 734 (5.2)	247 394 (1.3)
Total	458 227 687	423 116 633 (92.3)	26 549 705 (6.3)	20 496 994 (4.8)	11 788 616 (2.8)

## Discussion

Interpreting and understanding medication dose instructions relies upon knowing how much and how often a medicine is to be taken. These are elementary concepts, so we adopted a pragmatic approach in which we aimed to transform free-text dose instructions into regular structured information that could be readily used by research teams. This study, one of only a small number of published studies, reports our experience with the application of NLP approaches at scale to the NHS Scotland national prescribing dataset. Our study dataset comprised 458 227 687 prescriptions, of which 99.67% had dose instructions represented by 4 964 083 distinct texts; 13 593 (0.27%) of these occurred ≥1000 times, accounting for 405 743 493 (88.85%) of all the prescriptions with a free-text dose instruction. We developed an NLP algorithm which, on application to the study dataset, generated an overall structured output of 92.3% (ranging from 86.7% for CNS drugs to 96.8% for CVS drugs).

The application of NLP methods to support the interpretation of unstructured dose instructions, still commonplace in electronic prescribing systems, has the potential to significantly improve the efficiency of conducting drug utilization and pharmacoepidemiology studies; free-text dose instructions are cumbersome and can be difficult to interpret and analyse in large volumes. Transformation into structured dose attributes can enable calculations to be more easily performed to derive the intended daily dose, and to estimate the expected duration for which a prescription will provide treatment. This is the foundation on which further work can be performed to roll up prescribing events for different medicinal products containing the same active ingredient and combine records that overlap in time, subject to a persistence window, to generate measures to estimate adherence and drug eras to explore clinical outcome and safety.^15^^,^^16^

Our data model shares similarities with the approach used by Shah *et al*.^12^ and built upon by Karystianis *et al*.^7^ However, Shah *et al.* outputted a single numerical value for the amount to be consumed daily, with a flag to indicate whether this was a calculated average and if consumption was ‘as needed’, whereas Karystianis *et al*. outputted more granular information but converted the dose intervals to the number of times per day and set the minimum frequency to zero for ‘as required’ prescriptions. Both methods allow identification of variability within a dose instruction but lose elements of detail. In contrast, we have separated dose frequency information according to whether the instructions are based on a number of doses to be taken within a period (e.g. twice per day) or with an interval between each dose (e.g. every 4 hours), and separately present the variables ‘as required’ and ‘as directed’ as a qualifier attribute. This is consistent with our intention of developing a zero-assumption approach to our data provisioning.

The performance of our algorithm was validated through manual inspection by a multidisciplinary group, producing an accuracy of 94.2% (*n* = 13 152). This compares favourably to an accuracy of 98.8% (*n* = 1000) from Shah *et al*.^12^ and 90.9% (*n* = 220) from Karystianis *et al.*^7^ Unsurprisingly, our free-text dose instruction data include many of the issues previously identified by others, including misspellings, the use of acronyms and abbreviations and structural ambiguity.^7^ Some of these we have addressed by extending the lexicons used by the NLP algorithm, and others are resolved by the rule-set that looks for sequences or proximity of words to deduce meaning. However, our model remains challenged by complex dose instructions such as those that call for one dose to be taken for a period of time followed by a different dose (e.g. ‘one daily for 5 days and then one twice daily thereafter’). Our final algorithm is a balance between the drive towards dedicated rules to characterize individual distinct free-text dose instructions, and maintaining a manageable number of generic rules.

Our study reports, for the first time, analysis of the level of translation by individual therapeutic area, identified by Shah *et al.* as a limitation.^12^ The results reflect the complexity and flexibility in treatment regimens adopted in the management of acute and chronic disease. For example, cardiovascular disease had the highest translation rate (96.8%) and one of the lower rates for dosage flexibility (0.3%), affirming a level of dosage standardization in treatment. In contrast, CNS drugs, which include pain management therapy, illustrated the lowest translation rate (86.7%) and the highest dosage flexibility (21.3%), a consequence of individual dosage titration often over time in this therapeutic area. Furthermore, although the extent of use of qualifiers was relatively low, this also varied by therapeutic area. These data provide useful and important intelligence for researchers choosing to use real-world administrative datasets in their studies.

### Limitations

This study has a number of limitations. First, the model is dependent on the prescriber recording a dose instruction (99.67% in our sample) and including dose and frequency attributes (92.3%), to enable the algorithm to generate a structured output. Nevertheless, the present level of translation supports researchers to readily derive daily dose exposure for the majority of prescriptions, relying on manual interpretation and/or development of specific rules for any untranslated instructions. Second, we have built the NLP algorithm based on only systemic therapies covering BNF chapters 1–10. This largely omits topical and other non-oral therapies that account for 15% of prescriptions within the PIS dataset. However, it is likely that the algorithm would produce some structured output for these; but elements may be incomplete as prescribers often omit the quantity and frequency of each dose, using ‘as required’ and ‘as directed’ qualifiers. In these situations, researchers need to revert to quantity and frequency of supply to examine drug use. Finally, the design and validity of the algorithm are based predominantly on a sample of 15 539 distinct dose instructions which focused on the most frequently occurring instructions, and this is likely to explain the variation by therapeutic area (Table 5). However, clinical validation did include a second random sample from each frequency stratum in Table 2. Nevertheless, researchers should remain vigilant in undertaking quality checks throughout data transformation and analysis.

### Future direction

The algorithm is now (April 2017) in operation and researchers can request a free-text dose instruction translated output as part of their PIS data extract through eDRIS, NHS National Services Scotland [www.isdscotland.org/Products-and-Services/eDRIS/], the body that provisions national datasets on behalf of NHS Scotland. A 12-month review of the performance of the algorithm will be undertaken, including feedback from users of the output, and this will inform future algorithm versions. Preliminary feedback from two early studies examining methadone^17^ and direct oral anticoagulant therapy^16^ has been positive.

Furthermore, our algorithm was applied to dose instructions as recorded by GPs, so it should be applicable to and equally effective with data from other English language GP-based prescription datasets, such as the Clinical Practice Research database (CPRD)^18^ or The Health Improvement Network (THIN) database.^19^

## Conclusion

We have presented the successful adoption of a text-mining approach, through design and application of an NLP algorithm, as a route to the provisioning of large volumes of free-text dose instructions, generated through capturing all electronic prescriptions (about 100 million per annum) in primary care in Scotland. We have taken a zero-assumption approach to the codification and production of general rules to create the algorithm, ensuring that users of the data have maximum flexibility to formulate, test and apply their own assumptions according to the medicines, population and research questions under investigation. Data science expertise will become ever more important to assist the effective and safe management of ‘big data’, to enable rapid creation of new clinical knowledge for innovation in health services.
